# Docosahexaenoic acid and n-6 docosapentaenoic acid supplementation alter rat skeletal muscle fatty acid composition

**DOI:** 10.1186/1476-511X-6-13

**Published:** 2007-04-25

**Authors:** Ken D Stark, Sun-Young Lim, Norman Salem

**Affiliations:** 1 Laboratory of Nutritional and Nutraceutical Research, Department of Kinesiology, University of Waterloo, Waterloo, ON N2L 3G1, Canada; 2 Division of Marine Environment & Bioscience, Korea Maritime University, Busan 606-791, Korea; 3 Laboratory of Membrane Biochemistry and Biophysics, Division of Intramural Clinical and Biological Research, National Institute on Alcohol Abuse and Alcoholism, National Institutes of Health, Bethesda, MD 20892, USA

## Abstract

**Background:**

Docosahexaenoic acid (22:6n-3, DHA) and n-6 docosapentaenoic acid (22:5n-6, DPAn-6) are highly unsaturated fatty acids (HUFA, ≥ 20 carbons, ≥ 3 double bonds) that differ by a single carbon-carbon double bond at the Δ19 position. Membrane 22:6n-3 may support skeletal muscle function through optimal ion pump activity of sarcoplasmic reticulum and electron transport in the mitochondria. Typically n-3 fatty acid deficient feeding trials utilize linoleic acid (18:2n-6, LA) as a comparison group, possibly introducing a lower level of HUFA in addition to n-3 fatty acid deficiency. The use of 22:5n-6 as a dietary control is ideal for determining specific requirements for 22:6n-3 in various physiological processes. The incorporation of dietary 22:5n-6 into rat skeletal muscles has not been demonstrated previously. A one generation, artificial rearing model was utilized to supply 22:6n-3 and/or 22:5n-6 to rats from d2 after birth to adulthood. An n-3 fatty acid deficient, artificial milk with 18:2n-6 was supplemented with 22:6n-3 and/or 22:5n-6 resulting in four artificially reared (AR) dietary groups; AR-LA, AR-DHA, AR-DPAn-6, AR-DHA+DPAn-6. A dam reared group (DAM) was included as an additional control. Animals were sacrificed at 15 wks and soleus, white gastrocnemius and red gastrocnemius muscles were collected for fatty acid analyses.

**Results:**

In all muscles of the DAM group, the concentration of 22:5n-6 was significantly lower than 22:6n-3 concentrations. While 22:5n-6 was elevated in the AR-LA group and the AR-DPAn-6 group, 20:4n-6 tended to be higher in the AR-LA muscles and not in the AR-DPAn-6 muscles. The AR-DHA+DPAn-6 had a slight, but non-significant increase in 22:5n-6 content. In the red gastrocnemius of the AR-DPAn-6 group, 22:5n-6 levels (8.1 ± 2.8 wt. %) did not reciprocally replace the 22:6n-3 levels observed in AR-DHA reared rats (12.2 ± 2.3 wt. %) suggesting a specific preference/requirement for 22:6n-3 in red gastrocnemius.

**Conclusion:**

Dietary 22:5n-6 is incorporated into skeletal muscles and appears to largely compete with 22:6n-3 for incorporation into lipids. In contrast, 18:2n-6 feeding tends to result in elevations of 20:4n-6 and restrained increases of 22:5n-6. As such, 22:5n-6 dietary comparison groups may be useful in elucidating specific requirements for 22:6n-3 to support optimal health and disease prevention.

## Background

Docosahexaenoic acid (22:6n-3, DHA) demonstrates health benefits through various physiological systems including neurological [1], cardiovascular [2] and inflammatory systems [3]. These mechanisms are largely derived through 22:6n-3 incorporation in place of other fatty acids into biological membranes and cell signalling mechanisms. The fatty acid composition of muscle has been implicated in obesity and insulin resistance and dietary intake can modify these compositions [4,5]. Feeding rats an essential fatty acid deficient diet results in higher peak twitch tension, reduced twitch contraction and half-relaxation times and quicker fatigability as compared with n-6 and n-3 fatty acid enriched diets in soleus and/or extensor digitorum longus muscles [6].

The impact of dietary fatty acid manipulation on the fatty acid composition of rat skeletal muscle has been studied [7-10]. However, only one of these studies, examining a mixed muscle homogenate, [7] employed a traditional two generational dietary n-3 fatty acid deficiency model in which the dam and her offspring are raised on n-3 fatty acid deficient diets. This study demonstrated that n-6 docosapentaenoic acid (22:5n-6, DPAn-6) increased with deficiency, but the 22:5n-6 did not reciprocally replace lost docosahexaenoic acid (22:6n-3, DHA). The remaining studies introduced dietary fatty acid manipulation protocols post-weaning. The impact of fatty acid manipulation during early development is relevant given recent evidence demonstrating long-term physiological changes in the skeletal muscle of offspring with maternal nutrient restriction [11].

While it is possible to manipulate skeletal muscle 22:6n-3 levels with post-weaning dietary deficiency, it is known that other tissues, specifically those of the central nervous system are resistant to loss of 22:6n-3 with post-weaning deficiency [1]. In addition to two generational models, artificial rearing models have been recently employed for n-3 fatty acid and 22:6n-3 deficiency studies in the central nervous system [12-15]. In n-3 fatty acid deficiency studies, 22:6n-3 loss is often compensated to some degree by 22:5n-6 [7,16]. These highly unsaturated fatty acids (≥ 20 carbons, ≥ 3 double-bonds, HUFA) differ by only a single carbon-carbon double bond and are largely incorporated into the sn-2 position of phospholipids. Studies examining n-3 fatty acid deficiency often utilize a linoleic acid (18:2n-6, LA) based diet in comparison to a 22:6n-3 supplemented diet. Dietary 18:2n-6 may be beta-oxidized or incorporated into phospholipids, [17], but the utilization of 18:2n-6 for 22:5n-6 synthesis is very low [18] and 22:5n-6 does not completely replace 22:6n-3 concentrations in tissues such as the developing brain [16]. As such, 18:2n-6 feeding relies on elongation and desaturation activities to generate 22:5n-6 which typically results in increased in 20:4n-6 in addition to increased 22:5n-6.

Feeding 22:5n-6 to achieve reciprocal replacement of 22:6n-3 in muscle may be a novel and ideal control in functional muscle studies. Comparative anatomical studies have demonstrated a link between high frequency contraction and high levels of 22:6n-3 in muscles [19]. Both the sarcoplasmic reticulum and mitochondria of mixed muscle samples have been demonstrated to have high percentages of 22:6n-3 [20]. The fatty acid composition of skeletal muscle varies by muscle fibre type [21-24], however the content of 22:5n-6 is typically not reported. Dietary substitution of 22:6n-3 with 22:5n-6 has confirmed an important and specific role for 22:6n-3 in optimal neural function [12], but there is no data on dietary incorporation of 22:5n-6 into skeletal muscle. To our knowledge, there is only one other report of dietary 22:5n-6 feeding, however, the dietary oil used was also high in 22:6n-3 (10.2% 22:5n-6 and 35.7% 22:6n-3) [25].

The purpose of the present study is to determine if dietary 22:5n-6 is incorporated into rat skeletal muscle lipids after feeding with a 22:5n-6 oil free of 22:6n-3. The present study uses a unique artificially reared suckling rat model that allowed strict control of the type of fatty acids fed and included: a diet based on 18:2n-6 (AR-LA); and 18:2n-6 diets with either 1% 22:6n-3 (AR-DHA); 1% 22:5n-6 (AR-DPAn-6); or 1% 22:6n-3 plus 0.4% 22:5n-6 (AR-DHA+DPAn-6). A dam reared control group (DAM) was also included. The fatty acid compositions of total lipid extractions were determined for soleus (slow-twitch oxidative), red gastrocnemius (fast-twitch oxidative) and white gastrocnemius (fast-twitch glycolytic) muscles such that a measure of the total lipids in the muscles was obtained. Given the novel artificial rearing approach in the present study and the limited data on dietary 22:5n-6 supplementation, complete fatty acid compositions as detected, are reported rather than selected fatty acids.

## Results

### Body weight

The body weight of the rats in the present study have been presented previously [12]. Briefly, the AR groups showed lower weight gain than the DAM group until the time of weaning. There were no differences in body weight between AR and DAM groups by 8 weeks of age and at the time of sacrifice (15 weeks of age).

### Comparison of muscle types

Fatty acid compositions of total lipid extracts from soleus, red gastrocnemius and white gastrocnemius differed in relative weight percentages and concentrations in the dam-reared rats (Table 1). Total fatty acid concentrations were significantly higher in soleus as compared with red and white gastrocnemius which were similar. These differences were mainly a result of much higher concentrations of monounsaturates (over 3-fold higher than concentrations in red gastrocnemius and approximately 2-fold the white gastrocnemius concentration), but saturate and polyunsaturate (PUFA) concentrations in soleus were also significantly higher. Interestingly, concentrations of HUFA in soleus did not differ from white gastrocnemius and were significantly less than concentrations in red gastrocnemius. When fatty acids were expressed as relative weight percentages, the fatty acid composition of soleus tended to resemble white gastrocnemius, except for a significantly lower percentage of n-3 HUFA in total HUFA and total saturates. As compared to red gastrocnemius, soleus had similar total saturates, much higher total monounsaturates and lower levels of total PUFA and subclasses of PUFA including total HUFA.

**Table 1:** Relative Percentage and Concentrations of Sums and Classes of Fatty Acids in Soleus and Red and White Gastrocnemius Muscles from Dam-Reared Rats*

	**Soleus**	**Red Gastrocnemius**	**White Gastrocnemius**
	(weight % of total fatty acids)		
Total saturates	41.5 ± 0.8^a^	41.5 ± 1.2^a^	43.5 ± 1.3^b^
Total monounsaturates	28.7 ± 4.6^a^	17.4 ± 1.1^b^	24.4 ± 4.1^a^
Total PUFA	26.0 ± 4.8^a^	38.7 ± 1.6^b^	28.7 ± 4.8^a^
Total n-6 PUFA	20.7 ± 3.2^a^	25.4 ± 1.5^b^	19.4 ± 2.8^a^
Total n-3 PUFA	5.4 ± 1.6^a^	13.3 ± 1.1^b^	9.3 ± 2.1^c^
Total HUFA	11.5 ± 3.9^a^	23.3 ± 1.4^b^	16.2 ± 4.7^a^
% of n-3 HUFA in HUFA	39.1 ± 2.6^a^	55.8 ± 1.7^b^	54.1 ± 2.2^b^
			
	(concentration, μg/g)		
Total saturates	10828 ± 3784^a^	5520 ± 607^b^	6934 ± 1772^b^
Total monounsaturates	7813 ± 3881^a^	2317 ± 307^b^	3988 ± 1505^b^
Total PUFA	6441 ± 1159^a^	5130 ± 326^b^	4408 ± 473^b^
Total n-6 PUFA	5155 ± 1085^a^	3359 ± 206^b^	2995 ± 405^b^
Total n-3 PUFA	1285 ± 83^a^	1770 ± 209^b^	1413 ± 105^a^
Total HUFA	2711 ± 70^a^	3088 ± 294^b^	2425 ± 152^a^
Total fatty acids	26102 ± 9254^a^	13278 ± 1188^b^	15868 ± 3831^b^

*Values are means ± SD; n = 6. Values in a row not sharing a roman superscript are significantly different at *P *< 0.05 by Tukey's honestly significantly difference test after a significant *F*-value (*P *< 0.05) by repeated measures ANOVA. PUFA, polyunsaturated fatty acids; HUFA, highly unsaturated fatty acids (≥ 20 carbons, ≥ 3 carbon-carbon double bonds).

The fatty acid composition also varied at the level of individual fatty acids (Table 2). Although red gastrocnemius and soleus had similar weight percentage of total saturates (41.5 ± 1.15 and 41.5 ± 0.8 wt. %, respectively), red gastrocnemius had significantly lower 12:0 and 14:0 and significantly higher amounts of 16:0 and 18:0 as compared with soleus. White gastrocnemius resembled soleus in regards to 12:0, 14:0 and 18:0, but also had high levels of 16:0 (24.5 ± 1.0 wt. %). Other notable differences included significantly lower 16:1n-7 and 18:1n-9 in red gastrocnemius as compared with the others. Red gastrocnemius also had the highest percentage of 18:2n-6 (15.0 ± 1.3 wt. %) although not significantly different than soleus (13.6 ± 1.3 wt. %), and the highest percentage of 20:4n-6 (8.9 ± 0.44 wt. %) although not significantly different than white gastrocnemius (6.5 ± 2.2 wt. %). In regards to n-3 PUFA, red gastrocnemius had the lowest 18:3n-3, but the highest percentages of 22:5n-3 and DHA (11.7 ± 1.1 wt. %). DHA was the lowest in soleus (3.9 ± 1.6 wt. %) and intermediate in white gastrocnemius (7.7 ± 2.0 wt. %).

**Table 2:** Composition of Individual Fatty Acids in Soleus and Red and White Gastrocnemius Muscles from Dam-Reared Rats*

	**Soleus**	**Red Gastrocnemius**	**White Gastrocnemius**
	(weight % of total fatty acids)		
12:0	6.6 ± 2.3^a^	2.1 ± 0.8^b^	5.1 ± 1.9^a^
14:0	5.2 ± 1.1^a^	2.3 ± 0.4^b^	4.3 ± 1.2^a^
16:0 DMA	1.0 ± 0.5^a^	2.0 ± 0.4^b^	1.4 ± 0.4^ab^
16:0	19.4 ± 1.0^a^	21.7 ± 1.0^b^	24.5 ± 1.0^b^
18:0 DMA	0.53 ± 0.29	0.92 ± 0.25	0.75 ± 0.25
18:0	8.6 ± 2.4^a^	12.2 ± 0.6^b^	7.3 ± 1.0^a^
20:0	0.09 ± 0.02^a^	0.08 ± 0.02^a^	0.05 ± 0.01^b^
22:0	0.08 ± 0.02^ab^	0.09 ± 0.03^a^	0.05 ± 0.01^b^
24:0	0.13 ± 0.06^ab^	0.18 ± 0.02^a^	0.10 ± 0.02^b^
			
16:1 n-7	7.2 ± 1.8^a^	3.3 ± 0.3^b^	6.3 ± 1.5^a^
18:1 DMA	0.06 ± 0.02^a^	0.22 ± 0.05^b^	0.19 ± 0.06^b^
18:1 n-9	17.2 ± 2.7^a^	10.3 ± 1.0^b^	14.2 ± 2.7^a^
18:1 n-7	4.0 ± 0.3^a^	3.3 ± 0.2^b^	3.6 ± 0.2^b^
20:1 n-9	0.09 ± 0.03	0.06 ± 0.02	0.08 ± 0.02
24:1 n-9	0.12 ± 0.04^ab^	0.14 ± 0.01^a^	0.08 ± 0.02^b^
			
18:2 n-6	13.6 ± 1.3^a^	15.0 ± 1.3^a^	11.8 ± 0.8^b^
18:3 n-6	0.03 ± 0.01	0.03 ± 0.01	0.03 ± 0.01
20:2 n-6	0.06 ± 0.01	0.08 ± 0.05	0.05 ± 0.01
20:3 n-6	0.27 ± 0.07^a^	0.44 ± 0.05^b^	0.34 ± 0.09^ab^
20:4 n-6	6.0 ± 1.9^a^	8.9 ± 0.4^b^	6.5 ± 2.2^ab^
22:4 n-6	0.42 ± 0.09^a^	0.39 ± 0.03^a^	0.27 ± 0.09^b^
22:5 n-6	0.27 ± 0.11^a^	0.51 ± 0.07^b^	0.36 ± 0.17^ab^
			
18:3 n-3	0.82 ± 0.23^a^	0.34 ± 0.06^b^	0.62 ± 0.18^a^
20:5 n-3	0.07 ± 0.02^a^	0.13 ± 0.03^b^	0.10 ± 0.03^ab^
22:5 n-3	0.58 ± 0.19^a^	1.18 ± 0.07^b^	0.87 ± 0.24^c^
22:6 n-3	3.9 ± 1.6^a^	11.7 ± 1.1^b^	7.7 ± 2.0^c^

*Values are mean ± SD; n = 6. Values in a row not sharing a roman superscript are significantly different at *P *< 0.05 by Tukey's honestly significantly difference test after a significant *F*-value (*P *< 0.05) by repeated measures ANOVA. DMA, dimethyl acetal.

### Effect of dietary manipulations

There were no significant differences in the total concentration of all fatty acids with dietary treatment of each muscle; therefore comparisons of dietary treatments are restricted to relative weight percentages of total fatty acids. The percentage of 22:5n-6 was significantly higher in the AR-LA and AR-DPAn-6 groups in all muscles as compared to rats with dietary 22:6n-3, including the AR-DHA+DPAn-6 group. In fact, the percentage of 22:5n-6 in the AR-DHA+DPAn-6 group was not statistically different than either the DAM or the AR-DHA groups for all muscles. The potential ability of 22:5n-6 to replace 22:6n-3 in skeletal muscle was investigated by comparing the percentage of 22:5n-6 in the AR-DPAn-6 and AR-LA groups to the percentage of 22:6n-3 in the AR-DHA in each muscle. In soleus and white gastrocnemius, there were no differences, however in the red gastrocnemius, the amount of 22:5n-6 in the AR-DPAn-6 (8.1 ± 2.8 wt. %) and AR-LA (5.9 ± 1.9 wt. %) groups were significantly lower than the amount of 22:6n-3 in the AR-DHA group (12.2 ± 2.3 wt. %). While total HUFA was not significantly different in any of the muscles, the sum of 22:6n-3 + 22:5n-6 was significantly lower in the red gastrocnemius of the AR-DPAn-6 (9.2 ± 3.0 wt. %) and AR-LA (6.8 ± 2.1 wt. %) groups as compared to the AR-DHA group (13.5 ± 2.6 wt. %).

In the white gastrocnemius, the percentage of 20:4n-6 was significantly higher in the AR-LA group as compared with the AR-DHA+DPAn-6 group, with a tendency for a difference from the other groups as *P *< 0.10 for all individual comparisons (*P *= 0.098 versus DAM, *P *= 0.08 versus AR-DPAn-6, and *P *= 0.07 versus AR-DHA). Interestingly, the percentage of 20:4n-6 was the highest numerically, but not statistically different in the AR-LA groups of the red gastrocnemius and soleus. The percentage of 18:2n-6 was significantly higher in all muscles in the DAM group as compared to the AR rats. In white gastrocnemius, the AR-DPAn-6, AR-DHA+DPAn-6 and AR-DHA groups also had percentages of 18:2n-6 that were significantly lower than the AR-LA group. The total n-6 PUFA percentage in muscle tended to be lower in the AR-DHA+DPAn-6 and AR-DHA groups and higher in the AR-LA and AR-DPAn-6 groups, mainly as a result of the higher percentage of 22:5n-6. There was an exception in soleus as the highest observed percentage of total n-6 PUFA was in the DAM group because of a significantly higher percentage of 18:2n-6.

Total PUFA did not differ in red gastrocnemius and white gastrocnemius, but was significantly lower in soleus in the artificially reared as compared to the dam-reared rats (Tables 3, 4, 5). Total HUFA did not change with dietary fatty acid supplementation for any of the muscles. In all muscles, 22:6n-3 percentages were significantly lower in the 22:6n-3 free diets (AR-LA and AR-DPAn-6 groups) and the AR-DHA group had 22:6n-3 levels similar to the DAM group. In the AR-DHA+DPAn-6 group, 22:6n-3 tended to be lower in the muscles, but the only significant difference was in white gastrocnemius (5.4 ± 1.3 wt. % versus 7.7 ± 1.8 wt. % in dam reared). The DAM group had significantly higher 18:3n-3 and 22:5n-3 in all muscles, and significantly higher 20:5n-3 in red gastrocnemius and soleus as compared with the AR rats.

**Table 3:** Fatty Acid Composition of Rat Soleus After Dietary Supplementation with 22:6n-3 and 22:5n-6*

		**Artificially reared**			
	**Dam reared (n = 7)**	**AR-LA (n = 7)**	**AR-DPAn-6 (n = 7)**	**AR-DHA+DPAn-6 (n = 8)**	**AR-DHA (n = 7)**

Fatty acids	(weight % of total fatty acids)				
12:0	6.3 ± 2.2^a^	2.55 ± 0.46^b^	2.53 ± 0.45^b^	2.55 ± 0.70^b^	2.63 ± 0.73^b^
14:0	5.1 ± 1.1^a^	3.02 ± 0.27^b^	3.03 ± 0.25^b^	3.12 ± 0.40^b^	3.07 ± 0.51^b^
16:0 DMA	0.93 ± 0.42	0.75 ± 0.25	0.77 ± 0.37	0.66 ± 0.25	0.67 ± 0.36
16:0	20.1 ± 2.0	20.6 ± 1.6	20.8 ± 0.9	21.6 ± 1.0	20.3 ± 0.6
18:0 DMA	0.54 ± 0.27	0.26 ± 0.12	0.30 ± 0.17	0.28 ± 0.15	0.27 ± 0.15
18:0	8.4 ± 2.2	6.7 ± 1.7	6.6 ± 1.7	5.6 ± 1.4	6.4 ± 2.6
20:0	0.10 ± 0.02^a^	0.05 ± 0.02^b^	0.05 ± 0.01^b^	0.05 ± 0.02^b^	0.06 ± 0.03^b^
22:0	0.11 ± 0.08	0.04 ± 0.02	0.05 ± 0.02	0.05 ± 0.05	0.05 ± 0.03
24:0	0.15 ± 0.09^a^	0.06 ± 0.02^b^	0.07 ± 0.03^ab^	0.08 ± 0.06^ab^	0.09 ± 0.05^ab^
Total saturates	41.6 ± 0.8^a^	34.0 ± 2.1^b^	34.2 ± 2.4^b^	34.0 ± 1.6^b^	33.5 ± 2.1^b^
					
16:1 n-7	7.3 ± 1.6	7.8 ± 1.4	7.6 ± 1.0	8.3 ± 1.8	7.5 ± 2.3
18:1 DMA	0.08 ± 0.06	0.07 ± 0.02	0.08 ± 0.04	0.10 ± 0.11	0.08 ± 0.05
18:1 n-9	17.3 ± 2.5^a^	31.2 ± 3.7^b^	31.6 ± 4.2^b^	32.8 ± 3.8^b^	32.1 ± 5.5^b^
18:1 n-7	3.93 ± 0.36	4.55 ± 0.61	4.40 ± 0.50	4.27 ± 0.45	4.38 ± 0.33
20:1 n-9	0.09 ± 0.02^a^	0.19 ± 0.03^b^	0.18 ± 0.05^b^	0.20 ± 0.04^b^	0.20 ± 0.05^b^
24:1 n-9	0.13 ± 0.04	0.12 ± 0.04	0.11 ± 0.04	0.12 ± 0.05	0.14 ± 0.07
Total monounsaturates	28.8 ± 4.2^a^	44.0 ± 5.2^b^	44.0 ± 5.1^b^	45.8 ± 5.3^b^	44.4 ± 7.9^b^
					
18:2 n-6	13.1 ± 1.9^a^	8.4 ± 1.0^b^	8.24 ± 0.77^b^	7.82 ± 0.52^b^	8.4 ± 1.2^b^
18:3 n-6	0.03 ± 0.01^a^	0.03 ± 0.01^b^	0.024 ± 0.004^b^	0.022 ± 0.004^ab^	0.022 ± 0.004^ab^
20:2 n-6	0.06 ± 0.01^a^	0.04 ± 0.01^b^	0.04 ± 0.01^b^	0.04 ± 0.01^b^	0.05 ± 0.01^ab^
20:3 n-6	0.27 ± 0.06	0.22 ± 0.06	0.23 ± 0.07	0.20 ± 0.05	0.20 ± 0.07
20:4 n-6	5.8 ± 1.7	6.2 ± 1.8	5.4 ± 1.7	4.2 ± 1.4	4.9 ± 2.4
22:4 n-6	0.40 ± 0.09^ab^	0.56 ± 0.14^a^	0.41 ± 0.11^ab^	0.27 ± 0.03^b^	0.31 ± 0.14^b^
22:5 n-6	0.27 ± 0.10^a^	2.26 ± 0.73^b^	3.03 ± 0.99^b^	0.78 ± 0.29^a^	0.56 ± 0.32^a^
Total n-6 PUFA	19.9 ± 3.5^a^	17.7 ± 3.5^ab^	17.3 ± 3.2^ab^	13.3 ± 1.9^b^	14.4 ± 3.7^b^
					
18:3 n-3	0.78 ± 0.23^a^	0.03 ± 0.01^b^	0.023 ± 0.003^b^	0.03 ± 0.01^b^	0.04 ± 0.04^b^
20:5 n-3	0.08 ± 0.02^a^	*-*	*-*	0.02 ± 0.01^b^	0.025 ± 0.003^b^
22:5 n-3	0.59 ± 0.19^a^	0.04 ± 0.02^b^	0.04 ± 0.01^b^	0.06 ± 0.03^b^	0.08 ± 0.03^b^
22:6 n-3	4.3 ± 1.7^a^	0.32 ± 0.14^b^	0.44 ± 0.32^b^	3.2 ± 2.0^a^	3.7 ± 2.1^a^
Total n-3 PUFA	5.7 ± 1.7^a^	0.40 ± 0.16^b^	0.50 ± 0.32^b^	3.3 ± 2.0^c^	3.8 ± 2.1^ac^
					
Total PUFA	25.6 ± 4.5^a^	18.1 ± 3.7^b^	17.8 ± 3.4^b^	16.6 ± 3.9^b^	18.3 ± 5.7^b^
Total HUFA	11.7 ± 3.6	9.6 ± 2.8	9.5 ± 3.0	8.7 ± 3.6	9.8 ± 5.0
% of n-3 HUFA in HUFA	41.3 ± 6.3^a^	3.8 ± 0.6^b^	4.9 ± 2.7^b^	35.0 ± 6.9^a^	38.2 ± 3.12^a^
Total FA (mg/g)	26.1 ± 8.5	34.5 ± 10.6	37.4 ± 15.6	43.2 ± 17.2	40.0 ± 19.5

*Values are mean ± SD. Values in a row not sharing a roman superscript are significantly different at *P *< 0.05 by Tukey's honestly significantly difference test after a significant *F*-value (*P *< 0.05) by one-way ANOVA. AR-LA, 18:2n-6 artificial reared diet; AR-DPAn-6, 22:5n-6 artificial reared diet; AR-DHA+DPAn-6, 22:6n-3 + 22:5n-6 artificial reared diet; AR-DHA, 22:6n-3 artificial reared diet; DMA, dimethyl acetal; PUFA, polyunsaturated fatty acids; HUFA, highly unsaturated fatty acids (≥ 20 carbons, ≥ 3 carbon-carbon double bonds).

**Table 4:** Fatty Acid Composition of Rat Red Gastrocnemius After Dietary Supplementation with 22:6n-3 and 22:5n-6*

		**Artificially reared**			
	**Dam reared (n = 8)**	**AR-LA (n = 7)**	**AR-DPA (n = 7)**	**AR-DHA+DPA (n = 8)**	**AR-DHA (n = 7)**

Fatty acids	(weight % of total fatty acids)				
12:0	2.10 ± 0.84^a^	0.96 ± 0.53^b^	1.07 ± 0.76^ab^	1.00 ± 0.88^b^	0.84 ± 0.41^b^
14:0	2.44 ± 0.54^a^	1.62 ± 0.55^ab^	1.75 ± 0.69^ab^	1.60 ± 0.69^ab^	1.48 ± 0.48^b^
16:0 DMA	1.86 ± 0.44	1.37 ± 0.41	1.41 ± 0.46	1.71 ± 0.64	1.89 ± 0.62
16:0	22.1 ± 1.1^a^	22.5 ± 1.1^ab^	23.8 ± 1.5^b^	22.7 ± 0.8^ab^	22.2 ± 1.1^ab^
18:0 DMA	0.85 ± 0.26^a^	0.44 ± 0.19^b^	0.49 ± 0.16^ab^	0.57 ± 0.29^ab^	0.69 ± 0.29^ab^
18:0	11.9 ± 0.8	10.7 ± 2.1	9.9 ± 2.5	10.6 ± 2.3	10.5 ± 1.4
20:0	0.08 ± 0.02^a^	0.05 ± 0.01^b^	0.05 ± 0.02^b^	0.06 ± 0.01^b^	0.06 ± 0.01^ab^
22:0	0.08 ± 0.03	0.06 ± 0.01	0.05 ± 0.02	0.06 ± 0.02	0.08 ± 0.03
24:0	0.17 ± 0.02^a^	0.10 ± 0.02^bc^	0.09 ± 0.03^b^	0.13 ± 0.02^cd^	0.16 ± 0.03^ad^
Total saturates	41.6 ± 1.0^a^	37.8 ± 1.7^b^	38.6 ± 1.0^b^	38.4 ± 1.6^b^	37.9 ± 1.6^b^
					
16:1 n-7	3.44 ± 0.63	3.7 ± 1.8	4.1 ± 2.0	3.2 ± 1.2	3.19 ± 0.86
18:1 DMA	0.20 ± 0.06^a^	0.23 ± 0.09^ab^	0.24 ± 0.12^ab^	0.33 ± 0.14^ab^	0.39 ± 0.14^b^
18:1 n-9	10.6 ± 1.7^a^	18.6 ± 5.7^ab^	19.6 ± 7.1^b^	17.6 ± 7.0^ab^	17.7 ± 4.9^ab^
18:1 n-7	3.34 ± 0.19	3.70 ± 0.55	3.59 ± 0.30	3.70 ± 0.39	3.84 ± 0.40
20:1 n-9	0.07 ± 0.02^a^	0.13 ± 0.03^b^	0.14 ± 0.04^b^	0.12 ± 0.05^b^	0.11 ± 0.03^ab^
24:1 n-9	0.16 ± 0.04	0.15 ± 0.09	0.13 ± 0.05	0.18 ± 0.07	0.17 ± 0.03
Total monounsaturates	17.9 ± 2.3	26.5 ± 7.7	27.8 ± 9.1	25.2 ± 8.3	25.4 ± 5.6
					
18:2 n-6	14.5 ± 1.5^a^	12.1 ± 1.7^b^	10.3 ± 1.4^b^	10.2 ± 0.9^b^	10.3 ± 1.0^b^
18:3 n-6	0.03 ± 0.01^ab^	0.04 ± 0.01^a^	0.03 ± 0.01^ab^	0.03 ± 0.01^b^	0.03 ± 0.01^ab^
20:2 n-6	0.08 ± 0.05^a^	0.04 ± 0.01^b^	0.04 ± 0.01^b^	0.04 ± 0.01^b^	0.04 ± 0.01^ab^
20:3 n-6	0.43 ± 0.05	0.46 ± 0.12	0.43 ± 0.11	0.46 ± 0.15	0.42 ± 0.08
20:4 n-6	8.9 ± 1.0	11.8 ± 3.1	9.8 ± 3.8	9.2 ± 2.7	9.1 ± 1.5
22:4 n-6	0.38 ± 0.04^a^	0.92 ± 0.24^b^	0.62 ± 0.23^c^	0.39 ± 0.08^a^	0.37 ± 0.07^a^
22:5 n-6	0.53 ± 0.07^a^	5.9 ± 1.9^b^	8.1 ± 2.8^b^	1.97 ± 0.57^a^	1.30 ± 0.43^a^
Total n-6 PUFA	24.8 ± 2.2^abc^	31.3 ± 6.3^b^	29.3 ± 8.0^bc^	22.3 ± 3.8^ac^	21.6 ± 2.5^a^
					
18:3 n-3	0.35 ± 0.07^a^	0.02 ± 0.01^b^	0.02 ± 0.01^b^	0.03 ± 0.01^b^	0.02 ± 0.01^b^
20:5 n-3	0.13 ± 0.03^a^	*-*	*-*	0.03 ± 0.01^b^	0.04 ± 0.02^b^
22:5 n-3	1.18 ± 0.11^a^	0.11 ± 0.03^b^	0.09 ± 0.03^b^	0.17 ± 0.05^b^	0.19 ± 0.06^b^
22:6 n-3	11.6 ± 1.0^a^	0.89 ± 0.25^b^	1.14 ± 0.50^b^	11.0 ± 3.7^a^	12.2 ± 2.3^a^
Total n-3 PUFA	13.2 ± 1.1^a^	1.02 ± 0.27^b^	1.24 ± 0.51^b^	11.2 ± 3.7^a^	12.4 ± 2.4^a^
					
Total PUFA	38.1 ± 2.7	32.3 ± 6.5	30.5 ± 8.2	33.5 ± 7.3	34.0 ± 4.7
Total HUFA	23.1 ± 2.0	20.1 ± 5.4	20.2 ± 7.2	23.2 ± 4.2	23.6 ± 4.2
% of n-3 HUFA in HUFA	55.8 ± 1.8^a^	5.02 ± 0.30^b^	6.5 ± 2.8^b^	47.7 ± 3.3^c^	52.4 ± 3.0^a^
Total FA (mg/g)	13.5 ± 1.7	16.4 ± 6.5	16.7 ± 6.7	17.2 ± 8.1	14.3 ± 2.9

*Values are mean ± SD. Values in a row not sharing a roman superscript are significantly different at *P *< 0.05 by Tukey's honestly significantly difference test after a significant *F*-value (*P *< 0.05) by one-way ANOVA. AR-LA, 18:2n-6 artificial reared diet; AR-DPAn-6, 22:5n-6 artificial reared diet; AR-DHA+DPAn-6, 22:6n-3 + 22:5n-6 artificial reared diet; AR-DHA, 22:6n-3 artificial reared diet; DMA, dimethyl acetal; PUFA, polyunsaturated fatty acids; HUFA, highly unsaturated fatty acids (≥ 20 carbons, ≥ 3 carbon-carbon double bonds).

**Table 5:** Fatty Acid Composition of Rat White Gastrocnemius After Dietary Supplementation with 22:6n-3 and 22:5n-6*

		**Artificially reared**			
	**Dam reared (n = 7)**	**AR-LA (n = 7)**	**AR-DPA (n = 6)**	**AR-DHA+DPA (n = 8)**	**AR-DHA (n = 7)**

Fatty acids	(weight % of total fatty acids)				
12:0	5.0 ± 1.8^a^	1.50 ± 0.85^b^	1.98 ± 0.54^b^	2.08 ± 0.24^b^	1.93 ± 0.68^b^
14:0	4.3 ± 1.1^a^	2.11 ± 0.69^b^	2.68 ± 0.48^b^	2.73 ± 0.21^b^	2.49 ± 0.45^b^
16:0 DMA	1.35 ± 0.41	1.22 ± 0.55	0.95 ± 0.39	1.03 ± 0.25	1.17 ± 0.32
16:0	24.7 ± 1.0	26.0 ± 1.9	25.7 ± 0.4	25.9 ± 1.0	25.0 ± 1.2
18:0 DMA	0.71 ± 0.26^a^	0.41 ± 0.17^b^	0.35 ± 0.21^b^	0.36 ± 0.10^b^	0.43 ± 0.17^ab^
18:0	7.2 ± 0.9^a^	6.9 ± 1.7^ab^	5.7 ± 1.6^ab^	5.2 ± 0.8^b^	5.7 ± 1.5^ab^
20:0	0.05 ± 0.01^a^	0.04 ± 0.01^ab^	0.03 ± 0.01^b^	0.03 ± 0.01^b^	0.04 ± 0.01^ab^
22:0	0.05 ± 0.01^a^	0.04 ± 0.01^ab^	0.03 ± 0.01^b^	0.03 ± 0.01^b^	0.03 ± 0.01^ab^
24:0	0.10 ± 0.02^a^	0.06 ± 0.02^b^	0.05 ± 0.02^b^	0.06 ± 0.02^b^	0.08 ± 0.03^ab^
Total saturates	43.4 ± 1.3^a^	38.4 ± 2.5^b^	37.5 ± 1.0^b^	37.4 ± 0.9^b^	36.8 ± 1.3^b^
					
16:1 n-7	6.3 ± 1.4	5.4 ± 1.6	7.1 ± 2.0	7.0 ± 1.5	6.2 ± 1.9
18:1 DMA	0.18 ± 0.07	0.26 ± 0.08	0.21 ± 0.12	0.27 ± 0.08	0.30 ± 0.10
18:1 n-9	14.7 ± 2.8^a^	21.8 ± 7.3^ab^	27.0 ± 4.4^b^	27.6 ± 2.5^b^	25.9 ± 5.0^b^
18:1 n-7	3.60 ± 0.19^a^	3.78 ± 0.41^ab^	3.86 ± 0.25^ab^	4.04 ± 0.34^ab^	4.20 ± 0.34^b^
20:1 n-9	0.08 ± 0.02^a^	0.14 ± 0.05^b^	0.15 ± 0.05^b^	0.16 ± 0.02^b^	0.14 ± 0.03^b^
24:1 n-9	0.09 ± 0.04	0.10 ± 0.04	0.08 ± 0.03	0.10 ± 0.04	0.12 ± 0.05
Total monounsaturates	24.9 ± 4.0^a^	31.5 ± 8.8^ab^	38.4 ± 6.3^b^	39.1 ± 3.6^b^	36.9 ± 6.1^b^
					
18:2 n-6	11.6 ± 1.0^a^	10.0 ± 0.9^b^	8.1 ± 1.2^c^	7.7 ± 0.7^c^	8.3 ± 1.0^c^
18:3 n-6	0.033 ± 0.005^ab^	0.037 ± 0.003^a^	0.029 ± 0.004^bc^	0.025 ± 0.003^c^	0.027 ± 0.003^c^
20:2 n-6	0.05 ± 0.01^a^	0.04 ± 0.01^b^	0.03 ± 0.01^b^	0.03 ± 0.01^b^	0.03 ± 0.01^b^
20:3 n-6	0.34 ± 0.08	0.44 ± 0.16	0.32 ± 0.09	0.29 ± 0.07	0.31 ± 0.08
20:4 n-6	6.4 ± 2.0^ab^	9.8 ± 3.9^a^	6.2 ± 2.2^ab^	5.4 ± 1.3^b^	6.2 ± 2.2^ab^
22:4 n-6	0.27 ± 0.08^a^	0.81 ± 0.28^b^	0.42 ± 0.12^a^	0.24 ± 0.05^a^	0.25 ± 0.07^a^
22:5 n-6	0.37 ± 0.16^a^	4.5 ± 1.6^b^	5.0 ± 1.8^b^	1.17 ± 0.29^a^	0.81 ± 0.32^a^
Total n-6 PUFA	19.1 ± 2.7^a^	25.7 ± 6.7^b^	20.1 ± 5.1^ab^	14.9 ± 2.1^a^	15.9 ± 3.0^a^
					
18:3 n-3	0.61 ± 0.17^a^	0.02 ± 0.01^b^	0.018 ± 0.003^b^	0.03 ± 0.02^b^	0.02 ± 0.01^b^
20:5 n-3	0.10 ± 0.02	*-*	*-*	0.02 ± 0.01	0.02 ± 0.01
22:5 n-3	0.86 ± 0.22^a^	0.09 ± 0.04^b^	0.06 ± 0.02^b^	0.09 ± 0.02^b^	0.13 ± 0.05^b^
22:6 n-3	7.7 ± 1.8^a^	0.58 ± 0.25^b^	0.66 ± 0.40^b^	5.4 ± 1.3^c^	7.0 ± 2.1^ac^
Total n-3 PUFA	9.3 ± 1.9^a^	0.69 ± 0.28^b^	0.74 ± 0.41^b^	5.6 ± 1.3^c^	7.1 ± 2.1^ac^
					
Total PUFA	28.3 ± 4.5	26.4 ± 6.9	20.8 ± 5.4	20.5 ± 3.2	23.1 ± 5.0
Total HUFA	16.0 ± 4.3	16.2 ± 6.1	12.6 ± 4.4	12.6 ± 2.7	14.7 ± 4.6
% of n-3 HUFA in HUFA	54.2 ± 2.0^a^	4.1 ± 0.4^b^	5.7 ± 2.2^b^	43.8 ± 3.1^c^	48.6 ± 3.6^d^
Total FA (mg/g)	15.9 ± 3.5	16.3 ± 9.1	21.2 ± 7.6	21.1 ± 4.6	17.9 ± 6.3

*Values are mean ± SD. Values in a row not sharing a roman superscript are significantly different at *P *< 0.05 by Tukey's honestly significantly difference test after a significant *F*-value (*P *< 0.05) by one-way ANOVA. AR-LA, 18:2n-6 artificial reared diet; AR-DPAn-6, 22:5n-6 artificial reared diet; AR-DHA+DPAn-6, 22:6n-3 + 22:5n-6 artificial reared diet; AR-DHA, 22:6n-3 artificial reared diet; DMA, dimethyl acetal; PUFA, polyunsaturated fatty acids; HUFA, highly unsaturated fatty acids (≥ 20 carbons, ≥ 3 carbon-carbon double bonds)

Total n-3 PUFA percentages were significantly lower in the AR-LA and AR-DPAn-6 groups and similar for the DAM group and the AR-DHA group in all muscles. In white gastrocnemius and soleus, the total n-3 PUFA percentage in the AR-DHA+DPAn-6 group was significantly lower than the DAM group, but not the AR-DHA group. The AR-LA and AR-DPAn-6 groups had significantly lower percentages of n-3 HUFA in total HUFA than the other groups in all muscles. The response of this parameter to the inclusion of 22:6n-3 in the diet was not homogeneous for all three muscles. In soleus, there were no differences between the DAM, AR-DHA+DPAn-6 and AR-DHA groups. In red gastrocnemius, the DAM group (55.8 ± 1.8%) and AR-DHA group (52.4 ± 3.0%) were similar, but the AR-DHA+DPAn-6 group (47.7 ± 3.3%) had a significantly lower percentage of n-3 HUFA in total HUFA. In white gastrocnemius, the DAM, AR-DHA and AR-DHA+DPAn-6 groups were statistically different for each muscle at 54.2 ± 2.0%, 48.6 ± 3.6% and 43.8 ± 4.1%, respectively.

There were also differences observed in the saturated and monounsaturated fatty acids. Total saturates were significantly lower in all muscles of the AR rats as compared to the DAM group. This was largely driven by higher percentages in 12:0, 14:0, 20:0, 24:0 with a tendency for higher percentages of 18:0 and 22:0 in the DAM group muscles, while the percentage of 16:0 was largely similar. Total percentage of monounsaturates was lower in the DAM group, although the difference was not statistically different in the red gastrocnemius. This was mainly a result of significantly higher 18:1n-9 in all muscles.

## Discussion

The present study identifies a novel research method that can assist in determining the requirement of 22:6n-3 for proper skeletal muscle function including insulin resistance and diabetes. This study also confirms that dietary fatty acid intake can influence the fatty acid composition of muscle [7,9,10,26-28] and that the fatty acid composition of rat skeletal muscle differs significantly dependent on muscle fibre type [21-24]. The present study is the first to investigate the effect of 22:5n-6 supplementation via artificial rearing on the fatty acid composition of skeletal muscle and suggests that there may be subtle differences in how specific muscle fibres respond to dietary challenges.

In the present study, the percentage of total HUFA was not affected by the dietary manipulations although the amount of HUFA appears to be specific to each muscle type. There were obvious differences in the percentage of n-3 HUFA in total HUFA, as the diets without 22:6n-3 had markedly lower n-3 HUFA. The AR-LA group, which had no preformed dietary HUFA, did not differ however in total HUFA, and had the lowest percentage of n-3 HUFA and the highest 20:4n-6 wt. % in each muscle (although not statistically different than the AR-DPAn-6 group). Calculations for 80% power indicate that to determine significant differences (alpha = 0.05, two-tailed) between the percentage of 20:4n-6 in the AR-LA group and the AR-DPAn-6 group, n = 17, n = 31 and n = 53 would be required for each group for white gastrocnemius, red gastrocnemius and soleus, respectively. Although highly speculative, it appears that the AR-LA group may have had higher rates of elongation and desaturation of 18:2n-6 to 20:4n-6. Fatty acid isotope studies are required to verify this hypothesis.

The observation that 22:6n-3 levels are low in muscles when it is absent in the diet was expected. The 22:6n-3 levels presently observed in the AR-LA group (0.32 wt. % in soleus, 0.89 wt. % in red and 0.58 wt. % in white gastrocnemius) are in the same range as the 1.5 wt. % in phosphatidylethanolamine, 0.84 wt. % in phosphatidylserine + phosphatidylinositol and 0.14 wt. % in phosphatidylcholine reported by Tinoco et al. [7] and lower than post-weaning deficiency studies [8-10]. It appears that feeding 22:5n-6 with 22:6n-3 may have resulted in some competition for incorporation into muscle lipids, but the only significant difference was a lower 22:6n-3 wt. % in white gastrocnemius as compared to the DAM group. It appears that there is a preference for 22:6n-3 incorporation over 22:5n-6 into skeletal muscle. In the AR-DHA+DPAn-6 group, the diet provided 22:6n-3 and 22:5n-6 in a ratio of 2.5 to 1 while in muscle tissue composition it was 4.0 to 1 in soleus, 4.8 to 1 in white and 5.6 to 1 in red gastrocnemius. It was also observed that 22:5n-6 feeding did not result in a reciprocal replacement of 22:6n-3 in red gastrocnemius, possibly due to the high levels of 22:6n-3 in this tissue or possibly reflecting a highly selective mechanism for 22:6n-3 incorporation in red gastrocnemius. This ability to discriminate 22:5n-6 and 22:6n-3 has been shown in brain tissue in the same animals with the ratio of 22:6n-3 to 22:5n-6 in brain of 12.7 to 1 in the AR-DHA+DPAn-6 group. In the AR-DPAn-6 group, 22:5n-6 was 7.4 ± 0.3 wt. % and 22:6n-3 was 4.7 ± 0.3 wt. %, while in the AR-DHA group, 22:5n-6 was only 0.70 ± 0.27 wt. % and 22:6n-3 was 11.6 ± 0.4 wt. % [12].

Both 22:5n-6 and 22:6n-3 are HUFAs that are found predominantly in, and potentially compete for the *sn*-2 position of phospholipids. HUFA incorporation into neutral lipids such as triacylglycerols is often minor and undetectable as demonstrated recently in the rat [24]. The fatty acid content of lipid fractions of soleus, red and white gastrocnemius have been reported previously [21] with red gastrocnemius having the highest concentration of fatty acids in phospholipids and soleus having the highest concentration of fatty acids in triacylglycerols. In the present study, soleus muscle had the highest fatty acid content, but also the lowest percentage of total HUFA, although not significantly different than white gastrocnemius. In muscle, the sum of 16:0, 18:1n-9 and 18:2n-6 consists of approximately 75–85% of the total fatty acid content of the triacylglycerol fraction [21,24]. These three fatty acids can also be incorporated into phospholipids, but 18:1n-9 is predominantly found in neutral lipids. In the present study, the percentages of 18:1n-9, and 12:0 and 14:0 (also predominantly found in triacylglycerols) were significantly higher in soleus of the dam-reared rats.

The lipid class composition greatly influences the overall fatty acid composition of a tissue. In addition, the proportion of muscle membranes; the sarcolemma, sarcoplasmic reticulum and mitochondria, differ between muscle fibre types [29,30]. Mixed hind limb muscle preparations of sarcolemma, fragmented sarcoplasmic reticulum and mitochondria differ in fatty acid composition, which is not surprising given differences in polar and neutral lipid concentrations and ratios, neutral lipid composition, and phospholipid composition in these subcellular membranes [20,31,32]. In the presently analyzed muscle fibre types; white gastrocnemius fibres are the largest in diameter, have the highest amount of sarcoplasmic reticulum and the lowest mitochondria content, red gastrocnemius fibres are moderate in diameter, moderate in the amount of sarcoplasmic reticulum and have highest mitochondria content, and, soleus fibres are the smallest in diameter (therefore the highest sarcolemma content), have the lowest amount of sarcoplasmic reticulum and moderate mitochondria content.

The sarcolemma is unique in that the neutral lipid content is approximately equivalent to the phospholipid content and that neutral lipid composition approaches an even distribution between triacylglycerols, non-esterified fatty acids, cholesterol and cholesterol esters. In both the sarcoplasmic reticulum and mitochondria, phospholipids consist of approximately 80% of total lipids with triacylglycerol being the dominant neutral lipid and both fractions have similarly high percentages of 22:6n-3 (approximately 20%) [20]. Mitochondria have a higher percentage of phosphatidylethanolamine that has higher 22:6n-3 content than phosphatidylcholine [33]. However, mitochondrion also has higher levels of cardiolipin, which contains primarily 18:2n-6 and reportedly little to no DHA [31]. The significantly higher percentage of 18:0 in red gastrocnemius is also indicative of a higher proportion of phosphatidyl-ethanolamine in phospholipids [34]. The high percentage of 22:6n-3 observed in red gastrocnemius in the present study likely serves as structural cofactors to support fast oxidative activities [19].

Although exercise training has also been demonstrated to result in changes in fatty acid composition [4,8,35,36] and phospholipid molecular species [37], it is unlikely a factor in the differences observed in the present study. Motor activity was measured by a video image analyzer (Videomax V, Columbus Instruments, Columbus Ohio, USA) and presented previously [12]. Briefly, the AR-DPAn-6 group spent more time moving than the DAM, AR-DHA and AR-DHA+DPAn-6 groups, but there were no differences between the groups in total moving distance. Therefore, a training effect is unlikely and dietary manipulations are probably responsible for the observed differences in skeletal muscle fatty acids.

## Conclusion

Overall, the present study demonstrates that animals fed 22:5n-6 could be an important comparison group when examining the physiological impact of 22:6n-3. Feeding 22:5n-6 in comparison to feeding 22:6n-3 results in fatty acid composition changes that are largely restricted to 22:5n-6 and 22:6n-3 fatty acids. Feeding with 18:2n-6 feeding appears to increase 20:4n-6. This study also demonstrates that artificial rearing together with the use of semi-synthetic milk substitute is an effective method to manipulate skeletal muscle fatty acid compositions and to induce 22:6n-3 deficiency. The present study also demonstrates that muscle fibre type discrimination is important and that 22:6n-3 requirements may differ between fibre types, possibly in relation to sarcoplasmic reticulum and mitochondrial oxidation functions. It should be noted that the high percentage of 22:6n-3 observed in the in red gastrocnemius of the DAM group is comparable to levels found in brain in which 22:6n-3 has been demonstrated to have a critical role in supporting optimal function. The high level of 22:6n-3 in these muscles is believed to be a component of phospholipids that support optimal functioning of ion pump activity of sarcoplasmic reticulum and electron transport in the mitochondria as docosahexaenoic acid supports optimal G protein-coupled receptor signalling in retinal rod outer segments [38]. The deficiency and supplementation model using dietary 22:5n-6 as a comparison group as a presented here would assist in the elucidation of a specific role of 22:6n-3 in skeletal muscle physiology.

## Methods

### Animals and study design

A detailed description of the study design and experimental diets has been published previously [12]. All experimental procedures were approved by the Animal Care and Use Committee of the National Institute on Alcohol Abuse and Alcoholism, NIH. Briefly, pregnant, day 3 of gestation, Long-Evans rats were purchased (Charles River, Portage, MI, USA) and fed a 22:6n-3 free, but n-3 fatty acid adequate diet (3.1% of total fatty acids as α-linolenic acid, 18:3n-3). Animals were maintained in our animal facility with *ad libitum *water and at a controlled temperature (23 ± 1°C) and a 12-hr light/dark cycle. At postnatal day 2, male pups were collected from each litter and randomized to one of five experimental groups. The groups included a dam-reared group and groups artificially reared on one of four experimental milks. The composition of the experimental milks was based on an artificial milk with fat content from hydrogenated coconut oil (Dyets, Bethlehem, PA, USA), medium chain triglycerides (Mead Johnson Nutritionals, Evansville, IN, USA) and oleic and linoleic ethyl esters with purified docosapentaenoic n-6 and/or docosahexaenoic ethyl esters added (Nu-Chek Prep, Elysian, MN, USA). Thus, the five treatments included a dam-reared diet (DAM) where the dams received an n-3 fatty acid adequate (3.1% of total fatty acids as α-linolenic acid) but 22:6n-3 free diet, and four artificially reared (AR) groups. The four AR groups received the following diets: a 15% of total fatty acid 18:2n-6 based diet (AR-LA), the 18:2n-6 based diet with 1% of total fatty acids 22:5n-6 (AR-DPAn-6), the LA based diet with 1% of total fatty acids 22:6n-3 (AR-DHA) and a 18:2n-6 based diet with 1% of total fatty acids 22:6n-3 and 0.4% of total fatty acids 22:5n-6 diet (AR-DHA+DPAn-6). Details on the composition of the diets have been presented previously [12]. A summary of the fatty acid compositions as determined by gas chromatography are presented in Table 6. Pups were hand reared until they could feed *ad libitum *from water bottle nipples after eye opening (postnatal day 14–15) and were eventually weaned to a pelleted diet with a similar fatty acid composition with the DAM group transferred to the maternal diet. The male pups underwent a series of behavioral assessments as described previously [12] and were killed by decapitation. Soleus and gastrocnemius muscles were excised and red and white portions of the gastrocnemius muscles were separated, frozen on dry ice and stored at -80°C.

**Table 6:** Fatty Acid Composition of Artificial Rat Milk and Pelleted Rat Diets*

**Artificial Milk**	**Dam reared**	**AR-LA**	**AR-DPA**	**AR-DHA+DPA**	**AR-DHA**
Total saturates	n.d.	33.0	33.6	33.0	35.6
Total monounsaturates	n.d.	46.9	47.5	46.8	45.5
18:2 n-6	n.d.	18.1	16.0	17.7	16.1
18:3 n-3	n.d.	0.01	0.01	0.01	0.01
22:5 n-6	n.d.	-	1.01	0.42	-
22:6 n-3	n.d.	-	-	0.96	1.16
					
**Pelleted Diet**	**Dam reared**	**AR-LA**	**AR-DPA**	**AR-DHA+DPA**	**AR-DHA**

Total saturates	77.2	27.0	27.3	26.5	22.0
Total monounsaturates	4.3	46.2	44.5	44.7	47.1
18:2 n-6	15.3	15.4	15.1	15.3	16.3
18:3 n-3	3.1	0.04	0.04	0.04	0.05
22:5 n-6	-	-	1.04	0.49	-
22:6 n-3	-	-	-	0.98	1.10

*Values are weight % of total fatty acids. AR-LA, 18:2n-6 artificial reared diet; AR-DPAn-6, 22:5n-6 artificial reared diet; AR-DHA+DPAn-6, 22:6n-3 + 22:5n-6 artificial reared diet; AR-DHA, 22:6n-3 artificial reared diet; n.d., not determined.

### Analysis of muscle fatty acid composition

Total lipids were extracted from muscles by a modified Folch procedure [39] with docosatrienoic (22:3n-3) methyl ester (Nu-Chek Prep, Elysian, MN) included as an internal standard. Fatty acid methyl esters were prepared with 14% BF_3 _in methanol according to Morrison and Smith [40] with a modification to include hexane [41]. Fatty acid methyl esters were analyzed by gas chromatography as described previously [41].

### Statistical analysis

All statistical analyses were completed with SPSS for Windows statistical software (release 11.5.1; SPPS Inc., Chicago, IL). All data is expressed as mean ± standard deviation (SD). Comparisons between muscle types of dam-reared rats were completed by repeated measures ANOVA with each animal (n = 6) providing a complete set of the three muscle tissues. The effects of dietary treatment were compared by the General Linear Model procedure. Individual means were compared using Tukey's honestly significant difference test after a significant F-value was determined. Statistical significance was inferred at P < 0.05. The potential for dietary docosapentaenoic acid to replace docosahexaenoic acid was examined by *t *test comparisons of 22:5n-6 in the AR-DPAn-6 group with 22:6n-3 in the AR-DHA group.

## Competing interests

The author(s) declare that they have no competing interests.

## Authors' contributions

KDS contributed to the study design, assisted in animal rearing, performed muscle collection and fatty acid analyses and statistical analyses and was the primary author. SYL contributed to the study design, managed animal rearing, and contributed to manuscript revision. NS contributed to the study design, coordinated the study design and contributed to manuscript revision. All authors read and approved the final manuscript.
